# Physicians’ engagement in dual practices and the effects on labor supply in public hospitals: results from a register-based study

**DOI:** 10.1186/1472-6963-14-299

**Published:** 2014-07-10

**Authors:** Karl-Arne Johannessen, Terje P Hagen

**Affiliations:** 1 Sykehuspartner, PB 3572, 3007 Drammen, Norway; 2 Department of Health Management and Health Economics, University of Oslo, Oslo, Norway

**Keywords:** Physician dual practice, Labor supply, Public hospital, Gender differences, Norway

## Abstract

**Background:**

Physician dual practice, a combination of public and private practice, has attracted attention due to fear of reduced work supply and a lack of key personnel in the public system, increase in low priority treatments, and conflicts of interest for physicians who may be competing for their own patients when working for private suppliers. In this article, we analyze both choice of dual practice among hospital physicians and the dual practices’ effect on work supply in public hospitals.

**Methods:**

The sample consisted of 12,399 Norwegian hospital physicians working in public hospitals between 2001 and 2009. We linked hospital registry data on salaries and hospital working hours with data from national income and other registries covering non-hospital income, including income from dual work, cohabiting status, childbirths and socioeconomic characteristics. Our dataset also included hospital variables describing i.e. workload. We estimated odds ratio for choosing dual practice and the effects of dual practice on public working hours using different versions of mixed models.

**Results:**

The percentage of physicians engaged in dual practice fell from 35.1% for men and 17.6% for women in 2001 to 25.0% and 14.2%, respectively, in 2009. For both genders, financial debt and interest payments were positively correlated and having a newborn baby was negatively correlated with engaging in dual practice. Larger family size and being cohabitating increased the odds ratio of dual practice among men but reduced it for women. The most significant internal hospital factor for choosing dual practice was high wages for extended working hours, which significantly reduced the odds ratio for dual practice. The total working hours in public hospitals were similar for both those who did and did not engage in dual practice; however, dual practice reduced public working hours in some specialties.

**Conclusion:**

Economic factors followed by family variables are significant elements influencing dual practice. Although our findings indicate that engagement in dual practice by public hospital physicians in a well-regulated market may increase the total labor supply, this may vary significantly between medical specialties.

## Background

In health care systems that include both public and private providers, there may be competition for the recruitment of health care workers. Physicians who engage in ‘dual practice’ by working for both public and private care providers have attracted particular attention. Potential challenges described by the literature include reduced work supply and a lack of key personnel in the public system, an increase in low priority treatments, and conflicts of interest for physicians who may be competing for their own patients when working for private suppliers [1-6]. One theoretical model has predicted that reduced total health care delivery will result from physician dual practice [6]. Such hypotheses need to be empirically tested.

Physician dual practice is a term used to cover several differing aspects: physicians who combine work in the public system with work for private health care providers, education, research, management, or other economic activities not associated with health. In this paper, physician dual practice refers to physicians employed in public hospitals who also work in other types of health services, including ambulant emergency care where they substitute for private GPs (moonlighting), private not-for-profit hospitals or private for-profit hospitals and other providers.

Several reasons for dual practice have been suggested: a wide gap between physicians’ income expectations and wages in the public systems, long waiting lists and unsatisfactory working conditions in the public system, and other factors reflecting individual choices [1-3,7-16]. The reasons for dual practice may be quite diverse at the individual level, and an analysis of dual practice should recognize that individual employees might have quite different expectations and personal career goals.

Many of the empirical studies on this issue have been performed in countries with rather low public health salaries, and these studies may not reflect the situation in more developed countries where public salaries may be more competitive [16-20]. One of these studies [16] provided some support for the use of “rewarding” policies to retain physicians in the public sectors of more developed countries, while “limiting” policies are recommended for developing countries.

The Norwegian health system is mainly funded by general taxation. This also holds for the private sector, which consists of nonprofit hospitals with a well-defined catchment area, private for-profit hospitals specializing in day surgery, and specialists on contracts with regional health authorities. As a rule, GPs are organized as private practitioners on contract to municipalities. Private for-profit institutions also treat patients who pay out of pocket for their own care or are covered by voluntary health insurance.

There are no regulations in place that prohibit public employees from combining positions in public and private health care. In 1996, to possibly tackle a brain drain from public to private hospitals, a targeted increase in salaries for extended working hours and overtime work resulted in a substantial salary increase for physicians working in public hospitals. A study of that event indicated that the salary increase of 11% increased the physician labor supply to public hospitals and reduced physician dual practice [7]. Today, health care salaries in Norway compare favorably with salaries in the rest of the labor market, and per capita health care expenditure is among the highest in Europe [21].

A major reform in the Norwegian hospital sector was introduced in 2002, when all public hospitals in Norway were transferred from a system of county ownership to central government ownership [22]. Hospitals were restructured as health enterprises comprising 1–8 hospital units and organized within five regional health authorities (RHAs). In 2005, the number of RHAs was reduced from five to four. In the study period, the Norwegian hospital sector consisted of five regional university hospital enterprises (specialized hospitals), twelve central hospital enterprises (two with university functions), and eight local hospital enterprises. Regional hospitals, and to a certain degree central hospitals, use a substantial amount of their resources for research, whereas local hospitals mainly focus on patient care. Theoretically, this may influence both the workload and attractiveness of the institution. The aim of the 2002 hospital reform was to increase hospital efficiency by providing greater autonomy with respect to planning, budgeting, and workforce policies, and to ensure a more precise definition of their economic responsibilities. The reform occurred during a period in which private providers were building up their capacity in response to increasing waiting lists and waiting times. In 2002, the political signal was that RHAs should increase their use of private suppliers of specialist health care services to reduce increasing waiting lists. However, in 2005, a center-left coalition government announced its aim to reduce the use of private health care service providers. This resulted in a decrease in contracts between the RHAs’ and private suppliers.

### Aim and objectives

The aims of the current study are twofold. We first examine those factors that affect a physician’s decision to engage in dual practice. Second, we analyze how the engagement in dual practice affects the number of weekly working hours in public hospitals. We implement the analysis using a general model of physician labor supply where socioeconomic factors such as income and family structure and hospital specific factors such as physicians’ workload are allowed to affect dual practice and total working hours in public hospitals.

## Methods

### Study group

All 18,888 physicians who held a position in Norwegian public hospitals between 2001 and 2009 were considered for the study. We included physicians that had worked at least two years in public hospitals following graduation. To work as an independent physician in the private sector (and thus engage in dual practice), it is mandatory to be fully licensed as doctor, and this requires 18 months of intern service. Because of a lack of socioeconomic data, we excluded physicians who did not have a Norwegian social security number (usually short-term workers and those with incomplete data in public databases of social factors). Physicians above 67 years (the pension age) are excluded from the analyses. Using these criteria, the number of physicians in our sample ranged from 6,820 physicians in 2001 to 9,808 in 2009. In total, our sample consisted of 12,399 individual physicians.

### Data sources

We linked data from three different registers. Data of salaries and hospital working hours for each physician in each year (2001–2009) were obtained from The Employers Organization Specter, which annually reports such data to Statistics Norway. Data regarding nonhospital income and individual characteristics as cohabiting status and number of children were obtained for each physician in each year from Statistics Norway. To combine these two data sets we transferred the individual physician data from the Specter database to Statistics Norway, which linked the data and returned them as anonymous code.

Activity data for both the public hospitals and private providers consist of the total number of annual DRG activity for hospital stays, day treatments, and outpatient treatments. We recorded mean waiting times within each hospital region as variables reflecting the excess health care demand.

According to Norwegian regulations, the study did not require approval by The Norwegian Data Protection Authority or The Regional Committees for Medical and Health Research Ethics, but the study was subject to notification as required under the Norwegian Personal Data Act. Notification was submitted to the Norwegian Social Science Data Services (NSD) on 5 May 2008 (project number 19192).

### Variable definitions

We defined the two dependent variables as follows:

– *Dual practice*: In Norway, all income from all sources is registered annually for each individual. Nonhospital income is classified by Statistics Norway into the following categories: finance, teaching, moonlighting in primary care, salary from private health care, consulting/administrative work outside public hospitals, and salary from other nonhospital public service. Dual practice was described by a dummy variable taking the value of 1 if the physician received income as an individual person or as a private entrepreneur from moonlighting, private not-for-profit or private for-profit health care providers, and the value of 0 for all others.

– *Total weekly working hours:* the wage system for hospital physicians defines the standard basic weekly working hours to be 35.5–40.0 hours, which are fairly constant over time. In addition, doctors may voluntarily work extended hours each week on a regular basis (negotiated individually) and typically varying from 0 to 10 hours per week. Although these hours are usually stable over time, we recorded this variable each year for each individual physician. These two components make up the total planned working hours per week for each physician. Total weekly working hours also include casual overtime work, which may have monthly variations. Total weekly working hours describe the average total labor supply each week across the year.

The *salary for extended working hours* is based on national regulations and is calculated as 0.08% of the individual’s total regular annual salary. The hourly wage is related to position, experience, and education (e.g., PhD), and amounts to approximately twice the basic hourly wage. Individual attributes are described by the following variables; *Age*, *Gender* (men = 0, women = 1), being cohabitating (single, including divorced and widowed = 0, married, including formal partnerships according to Norwegian law = 1), *Children* < *18 years* (number of children under 18 years) and a dummy variable, *ChildLastYear*, indicating whether the individual had a child born (no child = 0, child born = 1) the previous year. Based on earlier analyses of significant gender effects on labor supply [23], we also analyzed the effects of gender on dual practice and therefore included interaction terms between gender and *Children* < *18 years*, *ChildLastYear*, and *cohabiting status*.

As indicated in the Background Section, previous reports have suggested that hospital characteristics may affect physicians’ decisions to engage in work in the private sector [7], and we therefore included hospital-specific variables. As an indicator of the workload (*Work Load*), we used the total number of diagnosis-related group (DRG) equivalents (the sum of DRG weights) per year from hospitalized patients and day treatment and outpatient visits divided by the total number of hospital beds to obtain the total DRG activity per hospital bed for each year. As a further indication of staffing, we calculated the number of FTE nurses and FTE physicians per hospital bed.

Individual-level economic data from Statistics Norway included total taxable income, total debt, total interest costs for loans, total income from savings, and income from nonhospital activity for each year. We calculated the net capital income *(NetCapInc)* as the sum of interest from savings and from total debt.

### Analytical approach and statistics

Between group analyses were done by Wilcoxon two-sample test.

The odds ratio of engaging in dual practice was analyzed using Generalized Linear Mixed Models (GLMM) with a binary response distribution and a logit link function. We used fixed effects for hospital and specialty to control for differences between specialties in some of the specifications. The statistical set up is inspired by previous economic analyses of physicians’ labor supply including those of Sloan, Noether, Rizzo and Blumenthal, and Baltagi et al. [24-27]. Our estimated models were derived from the life-cycle model and account for former income from work and capital by including lagged versions (values from previous year) of these variables. The regression equation of Dual practice (footprints for time are suppressed):

$$\begin{array}{c} \text{Dual}\;\text{practice}=a+a_{1}*\text{Salary}\;\text{for}\;\text{extended}\;\text{working}\;\text{hours}\left(\text{lag}\right)\\ \;+a_{2*}\text{NetCapInc}\left(\text{lag}\right)+a_{3*}\mathrm{IndAtt}+a_{4*}\mathrm{Hosp}\\ \;+a_{5*}\text{Waiting}\;\text{time}+u_{\mathrm{he}}+y+\mathrm{sp}+e \end{array}$$

where *NetCapInc* is the sum of capital income and expenses, *IndAtt* refers to individual characteristics such as gender, age, and cohabitating status. Age is categorized as follows: Age group1 < 35 years, 35 < = age group 2 < 45, 45 < = age group 3 < 55, 55 < = age group 4 < 68. Age group 4 serves as reference category. *Hosp* refers to hospital characteristics as *Workload, FTE physicians and Nurses per bed*. We included dummies (fixed effects) that were specific to each health enterprise (u_he_), year (y), and specialty (sp).

The regression equation for total weekly working hours:

$$\begin{array}{c} \mathrm{Total}\;\mathrm{Weekly}\;\mathrm{Working}\;\mathrm{Hours}=b+b_{1}*\mathrm{Salary}\;\mathrm{for}\;\mathrm{extended}\;\mathrm{working}\;\mathrm{hours}\left(\mathrm{lag}\right)\\ \;+b_{2*}\mathrm{NetCapInc}\;\left(\mathrm{lag}\right)+b_{3*}\mathrm{IndAtt}+b_{4*}\mathrm{Hosp}\\ \;+\mathrm{DualPractice}+u_{\mathrm{he}}+y+\mathrm{sp}+e, \end{array}$$

where *DualPractice* describes whether the physician has dual practice or not (dual practice = 1, no dual practice = 0).

The fixed-effects analyses imply that we utilized the variation between individuals within each health enterprise, year, and specialty. We assumed all remaining errors to be white noise (e). Dummies for health enterprises will, for example, capture the effect of the hospital hierarchy. We analyzed the dataset as unbalanced.

We used SAS software version 12.4 (SAS Institute, Cary, NC).

## Results

### Dual practice

**Figure 1:**
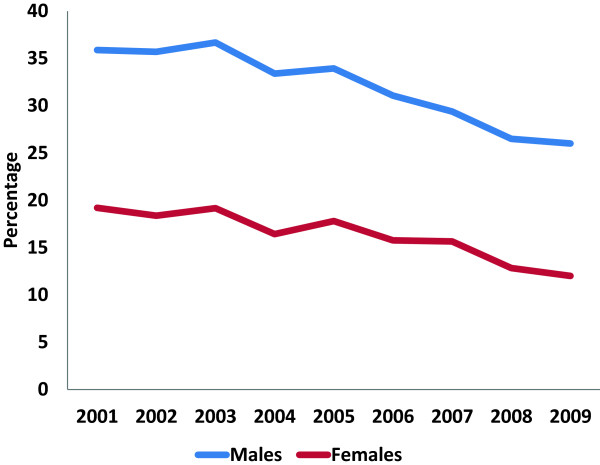
Percentage of male and female physicians engaged in dual practice, 2001–2009.

During the study period, twice as many men as women performed dual practice. There was a steady decline in the percentage who engaged in dual practice, from 35.1% of male physicians and 17.6% of female in 2001 to 25.0% and 14.2% in 2009, respectively (Figure 1).

**Figure 2:**
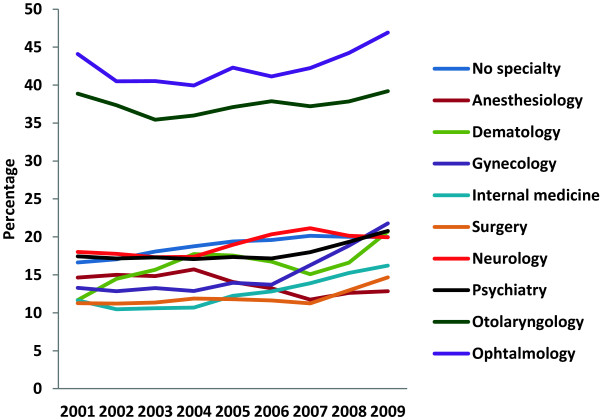
Earnings from dual practice as a percentage of physicians’ total annual income in selected specialties.

The incomes from dual practice varied considerably between the specialties; incomes from dual practice are illustrated as a percentage of physicians’ total income in some of the specialties with the highest dual practice payments in Figure 2. Only physicians with income from dual practice are included. The figure illustrates that otolaryngology and ophthalmology stand out with especially high relative payments, being approximately twice the level of the other specialties. The incomes from dual practice for those engaging in such practices increases at the same time as the share of physicians in dual practice are reduced (Figure 1 and Figure 2). Further descriptive statistics are given in Table 1.

**Table 1:** Descriptive statistics for the study groups with and without dual practice

	**Without dual practice**			**With dual practice**			**P-val nondual versus dual**
**Variable**	**Median**	**Maximum**	**Minimum**	**Median**	**Maximum**	**Minimum**	
Age	43	77	24	45	74	24	
Total debt	771 751	28 633 481	0	1 123 071	27 643 218	0	<0.01
Net capital income	-35 026	9 455 296	-7 042 827	-52 606	9 972 644	-3 912 604	<0.01
Percent females	44.6%			25.3%			<0.01
Percent of group who is cohabiting	76%			87%			<0.01
Number of children < 18 years (mean)	1	9	0	1	7	0	
Child born last year (mean)	0.07	1	0	0.05	1	0	<0.01
Average of full time equivalent	100	120	20	100	100	20	
Mean extended hours/week	5.6	74.9	0	5.6	70	0	
Average overtime hours/week	2.2	36.0	0	2.0	31.52	0	
Total working hours	44.2	96.58	0	44.1	90	0	
Median age in age group 1	32			31			
Median age in age group 2	39			40			
Median age in age group 3	49			49			
Median age in age group 3	59			59			
DRG per hospital bed	109	186	54	108	186	29	
FTE physicians per hospital bed	0.62	1.11	0.17	0.61	1.11	0.3	
FTE nurses per hospital bed	1.9	4.4	2.0	1.9	4.4	2.0	
Regional waiting time for patients	62	103	47	56	103	47	

### Odds ratio for choosing dual practice

Females (*Gender*) had a significantly lower odds ratio for choosing dual practice (p < 0.001) in all our analyses (Table 2). Having a child born last year reduced the odds ratio for both genders, although it was strongest for females. Being cohabiting and having a higher number of younger family members increased the odds ratio for men, but was negative for women. More debt and interest costs (negative values of net capital income) increased the odds ratio of choosing dual practice significantly for both genders. In the fixed effect analysis, senior consultants had a slightly higher odds ratio for dual practice than residents, and dual practice was highest in the oldest physician age group.

**Table 2:** Engagement in dual practice 2001-2009 (Odds Ratio with 95% Confidence Interval)

	**Model 1**		**Model 2**	
	**Odds ratio**	**Confidence interval**	**Odds ratio**	**Confidence interval**
Salary for extended work hours (lag)	0.999	0.999 - 0.999	1.000	0.999 - 1.000
Net capital income (lag) ( 10 000 NOK)	0.963	0.961 - 0.966	0.964	0.961 - 0.967
Gender (female=1)	0.697	0.652 - 0.744	0.697	0.652 - 0.746
Cohabiting	1.074	1.024 - 1.128	1.112	1.058 - 1.168
Cohabiting by gender	0.710	0.653 - 0.772	0.681	0.626 - 0.741
Number of children<18	1.007	0.987 - 1.028	1.015	0.995 - 1.037
Number of children<18 by gender	0.883	0.853 - 0.915	0.879	0.848 - 0.911
ChildLastYear	0.877	0.797 - 0.965	0.878	0.796 - 0.968
ChildLastYear by gender	0.571	0.479 - 0.682	0.562	0.470 - 0.672
Age group 1	1.016	0.941 - 1.098	0.938	0.867 - 1.016
Age group 2	0.667	0.626 - 0.711	0.646	0.605 - 0.689
Age group 3	0.952	0.901 - 1.006	0.937	0.885 - 0.991
Work load	1.113	1.006 - 1.232	0.962	0.789 - 1.174
Physician group	0.991	0.937 - 1.048	1.081	1.019 -1.147
Intercept	0.343	0.291 - 0.404	0.073	0.055 - 0.095
Year (fixed effects)	Yes		Yes	
Specialty (fixed effects)	No		Yes	
Health enterprises (fixed effects)	No		Yes	
Akaike [28] information criterion	75039.35		72885.15	
Pearson’s Chi-Square	67842.09		67717.30	
N	12399		12399	

The most striking hospital variable that correlated to the choice of dual practice was the wage level for extended work in the public hospitals, which reduced the odds ratio significantly.

From 2005 to 2007, the mean waiting times increased by 10%–35% in the differing regions. However, the extent of dual practice fell in the same period and there were no correlation between waiting times and odds ratio for dual practice in the multivariate analysis. We experimented with several combinations of the hospital variables as well as forward stepwise analyses (‘smaller is better’). Staffing indicators (physicians and nurses per bed) and waiting lists did not correlate to the odds ratio. An apparent increasing effect on the odds ratio of DRG per hospital bed was eliminated when using fixed effect analysis (Table 2).

### Working hours in public hospitals

**Figure 3:**
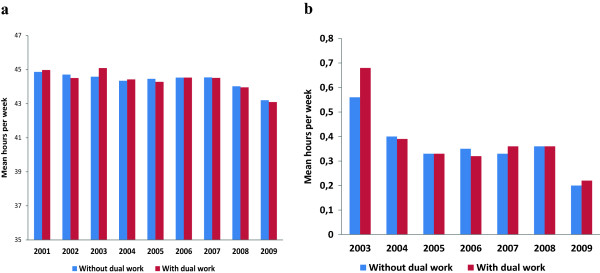
Mean planned working hours (a) and mean overtime hours per week (b) among physicians with and without dual practice (2001–2009).

Annual mean planned working hours, the mean number of overtime hours and hours on call (data not shown) in public hospitals were similar between groups with and without dual practice in the bivariate comparisons (Figure 3). Accordingly, total working hours per year were also similar. This was also confirmed in the regression analyses where engaging in dual practice did not reduce total working hours (Table 3).

**Table 3:** Total weekly working hours in public hospitals

	**Model 1**		**Model 2**	
	**Estimate**	**Confidence interval**	**Estimate**	**Confidence interval**
Salary for extended work hours (lag)	-0.002	-0.002 - -0.001	-0.001	-0.001 - 0.000
Net capital income (lag) (NOK 10 000)	-0.026	-0.036 - -0.015	-0.033	-0.043 - -0.023
Gender (female=1)	-1.133	-1.356 - -0.910	-0.885	-1.104 - -0.667
Cohabiting	-0.441	-0.618 - -0.265	-0.262	-0.435 - -0.090
Cohabiting by gender	-0.584	-0.853 - -0.315	-0.432	-0.694 - -0.170
Number of children <18	-0.254	-0.328 - -0.181	-0.263	-0.334 - -0.191
Number of children <18 by gender	-0.364	-0.473 - -0.255	-0.399	-0.505 - -0.292
ChildLastYear	0.688	0.342 - 1.035	0.784	0.447 - 1.121
ChildLastYear by gender	-1.710	-2.204 - -1.216	-2.077	-2.558 - -1.597
Age group 1	4.602	4.339 - 4.865	4.101	3.838 - 4.364
Age group 2	1.811	1.594 - 2.028	1.948	1.731 - 2.164
Age group 3	1.190	0.993 - 1.386	1.251	1.054 - 1.447
DRG per hospital bed	1.831	1.482 - 2.179	1.157	0.508 - 1.806
Dual practice	-0.159	-0.297 - -0.021	-0.126	-0.262 - 0.010
Physician group	-2.650	-2.833 - -2.467	-1.933	-2.117 - -1.749
Intercept	-2.650	-2.833 - -2.467	-1.933	-2.117 - -1.749
Year (fixed effects)	Yes		Yes	
Specialty (fixed effects)	No		Yes	
Health enterprises (fixed effects)	No		Yes	
Akaike [28] information criterion	470679.5		466800.6	
N	12399		12399	

The wage coefficients were negative but close to zero, indicating that the general wage increases over time had no effects on the labor supply in the period analyzed. As already indicated, there were no general significant effects of dual practices and total weekly working hours in public hospitals. However, separate analyses for otolaryngology and ophthalmology, the two specialties with the highest levels of non-public income, revealed a stronger relationship between dual practice and working hours in public hospitals, with estimates in the range of 3–5 fewer hours a week in both specialties. There were no significant interaction effects between dual practice and gender (estimates not shown), indicating that dual practice affects the labor supply in public hospitals similarly in both genders.

Further effects are in line with former analyses [23]. Women worked fewer hours than men did. After controlling for other variables, the effect was approximately 0.88 hours per week. The effects of children diverged strongly between the genders. In general, a child born last year increased the working hours among male physicians and substantially reduced it among females.

## Discussion

To our knowledge, this register-based study of 12,399 physicians in Norway is one of the largest to investigate physician engagement in dual practice in a fairly systematic setting including both genders. Furthermore, our study period of nine years seems to be one of the longest.

We found that approximately twice as many men as women participated in dual practice. Our study was conducted within a system that has not banned or regulated dual practice. Nevertheless, the proportion of physicians engaging in dual work declined by approximately 30% during the study period. The reason for this may be related to several factors. First, increasing hourly rate for extended working hours in hospitals reduced the odds ratio for engagement in dual practice significantly. This salary rate was instituted in 1996 specifically to increase physicians’ willingness to work extended hours to ensure a sufficient labor force in hospitals. Our findings may indicate that the effect of this action has been successful and is lasting over time. It supports the findings reported in other Norwegian studies [7,29]. Furthermore, the general regulations reducing the hours of work of Norwegians in recent decades may also be of significance.

Our analyses indicated that dual workers overall provided a similar labor supply to public hospitals as their non-dual worker colleagues, with respect to the planned working hours, on call duties, and overtime work. Thus, although our results may seem to contradict the hypothesis that dual workers reduce their public labor supply and avoid engagement in variable work and overtime [6,18], they are in accordance with previous British and Danish studies [9,30]. In fact, in our study, physicians engaged in dual work seemed to provide more than their fair share of the physician labor supply, which has also been suggested in other reports [3,9,10,20,30].

However, in particular specialties such as otolaryngology and ophthalmology, we found significant higher dual work incomes than in other specialties, and in these fields, the dual workers definitely worked less in the hospitals than their non-dual working colleagues. This significant variation between differing specialties may be associated with several facts. Our main hypothesis, however, is that financing for private services in these two specialties has been particularly abundant. This was instituted long before our study period as a governmental response to persistently long waiting lists for these patient groups over the decades.

### Individual attributes influencing choice of dual practice

**Figure 4:**
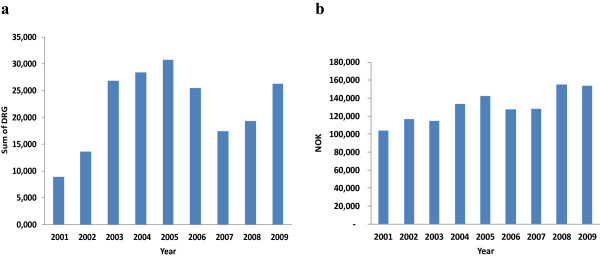
Sum of DRG activity at private hospitals (a), and mean earnings per physician from dual practice (b) (2001–2009. 1 NOK = $6.5 in 2009).

Economic factors seem to be one of the most important motives for choosing to engage in dual practice, even in a well-salaried system as found in Norway. The dual practitioners had higher levels of debt and thus more negative net capital income than their non-dual practicing colleagues did, and this was the most important correlation after gender and children. Our finding that having a child in the last year significantly reduced dual practice for females is not surprising as childbirth, for obvious reasons, has a larger impact on the lives of women than men. Furthermore, the finding that having a larger family size has a greater negative impact on dual practice for women probably indicates that domestic duties are still largely considered to be the responsibility of women.Our observation of a considerable variation in the extent of dual practice that paralleled the changes in private services between 2001 and 2009 may indicate that dual practice may be influenced by health marked conditions (Figure 4). The finding of higher odds ratio among the consultant and senior physicians is as expected, as there are more specialists among those holding these positions. The significance of the slightly higher odds ratio in the age group above 55 years is unclear, but needs further investigation.

Previous reports have indicated that unfavorable hospital characteristics may contribute to physicians choosing dual practice [7-11]. Several of these studies focused on special medical fields. In our study, higher levels of physician or nurse staffing per hospital bed did not influence the odds ratio to choose dual practice. One interpretation may be that the effects of such hospital factors are weak determinants of dual practice in a system like the Norwegian, where public hospitals have fair conditions regarding equipment, technology and staffing. Furthermore, such factors may be less important than economic factors and motives in other health care systems. Our main conclusion is, however, that factors influencing dual practice, both individual and hospital factors, are rather complex and should be analyzed in an appropriate context and not separately.

### Other aspects of dual practice

The literature is rather inconclusive regarding the differing aspects of dual practice, probably because of a lack of solid data. Negative implications have been proposed for general health care costs, the availability of health services, waiting lists, and the quality of public services [1,2,13,19,31,32]. Several of these factors are challenged by our results. Except for two specific specialties, the dual practitioners in our study worked just as many hours as their colleagues in public hospitals. Furthermore, the incidence of dual practice fell in the first two years after the 2005 policy change, even though waiting times increased. Neither was it found that the substantial variation in the extent of dual practice in the period was associated with a corresponding variation in public working hours. Thus, we find little support for the theoretical model published in 2007 [6] that claimed a reduction in total health services due to dual practice.

Several reports have proposed that dual practice should be restricted and regulated. Such steps have been exercised in several countries, but bans in the form of exclusive public sector contracts have rarely been successful [2,5,17,33-37]. Intermediate steps have been taken, for example, in the UK and France, where public specialists are allowed to earn 10% (UK) or 30% (France) of their total income from private fees [17,38]. However, a study from 2003 reported that despite such regulations, National Health Service consultants on average had a private income of 26% of their NHS income [5]. Except for otolaryngology and ophthalmology, which had average dual incomes of 35%–45% of the total income in our study, the other dual practitioners in our study had more moderate dual earnings of less than 20% of their total incomes (Figure 2).

There is a wide cross-national heterogeneity in the extent of dual practice [12]. Furthermore, there is a consensus in the literature that countries with lower public salaries may be more prone to experience dual practice than countries where salaries are competitive. Our results must be considered in the context of a well-resourced Norwegian public system. In our study, we observed that less than 30% of physicians engage in dual practice in Norway, which is lower than the rate found in most international studies. It is also lower than previously reported among Norwegian physicians [7]. This may be because in Norway, doctors’ public salaries are competitive. This may indicate that the effect of the salary adjustments made in 1996 has been preserved during follow-up negotiations for physician salaries, and that such mechanisms work.

In both the EU and US, a significant reduction in working hours should be expected over time with the implementation of the European Working Time Directive and the 2011 Accreditation Council for Graduate Medical Education duty hour standard, respectively [39,40]. Whether this also will influence dual work remains to be seen.

**Figure 5:**
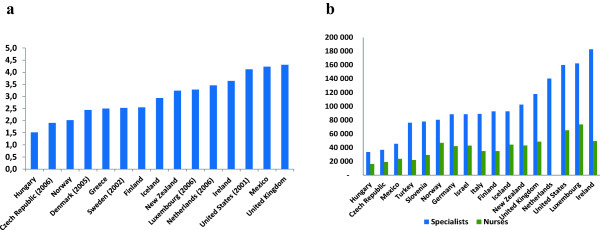
(a) Physician specialist’s annual salary, ratio to average salary of population (2007 or latest year available), and (b) annual salary of specialist doctors and nurses (2008. USD $).

Most previous reports have studied dual practice in health care systems that are quite different to the Norwegian system, which is a largely publicly financed system characterized by the strong regulation of patient rights and the labor rights of health care workers. For example, regulations concerning the hours of work of Norwegians have considerably reduced annual working hours in recent decades. Thus, our Norwegian results may refer to a framework that is somewhat advanced compared with most other health care systems with respect to lower working hours and widely available (and desirable) social benefits. Comparing dual practice across differing national systems is challenging. It may be argued that our results from Norway (which is a high-cost country with rather high salary levels as compared with most other European and Western countries) are influenced by a remuneration system for Norwegian hospital doctors that is too favorable to be relevant to other health care systems. However, the compensation received by Norwegian physicians is not too different from that in other countries when living costs and salaries of other workers are considered. Figure 5 illustrates that physician remuneration compared with average wages and nurses is no more favorable in Norway than in other systems [41].

### Limitations

Our register-based study did not include individual variables of motivation, work satisfaction, and career goals. Therefore, several factors that may influence physicians’ choices to engage in dual practice were not included in our study.

The criteria for including other income as dual practice may differ between studies. Whereas we captured all taxable income from non-public clinical work, this may vary between countries. We focused on dual medical practice and did not include income from nonmedical activities.

Analyzing hospital characteristics across multiple specialties means sacrificing specificity for breadth, and this applies to all our hospital variables. DRG equivalents are not an exact measure of patient-related workload because they include compensation for non-personnel routine operating costs attributable to patient care, medical and technical tools, routine nursing services, and so on. Furthermore, inpatient and outpatient DRG metrics must also be pooled with care because inpatient and outpatient activities consume quite different amounts of physician labor. In addition, some specialties, such as pathology and laboratory medicine, are involved in quite different ways than the specialties related to direct patient contact. Another issue is that DRG measures do not reflect research, education, and several other work duties. We cannot rule out that more specific characteristics of hospitals could have given different regression results. Nevertheless, DRG measures are the standard official measures of treatment activity in the annual governmental reports that assess productivity in Norwegian hospitals today.

We cannot ignore the fact that more detailed and specific measures of hospital staffing than we used for nurse and physician staffing could have given other correlates to the odds ratio of choosing dual practice, and such variables should probably be refined.

## Conclusions

We conclude that dual practice seems to be strongly related to economic motives, even in a system with competitive salaries in the public system, and that it has a dynamic nature that is responsive to the financial incentives offered. The gender differences that have been previously described in other workforce-related perspectives also seem to be reflected in dual practice, with family and socioeconomic factors as strong determinants, even in a system with well-developed equity policies. We also conclude that although dual work does not seem to reduce working hours in the public system from an overall perspective, it may definitely influence the public workforce in specific fields of medicine. The most important corollary therefore seems to be that care should be taken when making general conclusions regarding dual practice based on studies of particular medical fields. And vice versa, conducting studies on a macro level may miss particular specialties of interest and concern. Our conclusion is that dual practice should be analyzed in a broad context and not from a narrow perspective. Furthermore, there is obviously large diversity between differing countries and health systems. As health care delivery in the future may face a substantial workforce shortfall, it may be argued that the sector cannot afford to lose any kind of labor supply, and in this respect, policymakers may have to pursue different objectives depending on the actual health care system. The heterogeneity of dual practice suggests that it should not be debated as a polarized problem between public and private health care, but should be evaluated from an analytical viewpoint and based on facts [22,35,36].

## Competing interests

As authors we declare that we have no competing interests with respect to this publication.

## Authors’ contributions

KAJ gathered the data, analyzed the general statistics and drafted the main manuscript. TPH conducted the mixed model analyses and contributed significantly to the drafting of the manuscript. Both authors have approved the final manuscript.

## Pre-publication history

The pre-publication history for this paper can be accessed here:

http://www.biomedcentral.com/1472-6963/14/299/prepub

## References

[B1] Iversen T The effect of a private sector on the waiting time in national health service J Health Econ 1997 14 4 381 396 10.1016/S0167-6296(96)00518-8 10169097

[B2] Gonzalez P Should physicians’ dual practice be limited? An incentive approach Health Econ 2004 14 6 505 524 10.1002/hec.890 15185383

[B3] Gonzalez P On a policy of transferring public patients to private practice Health Econ 2005 14 5 513 527 10.1002/hec.946 15497171

[B4] Cullis JG Jones PR Propper C Anthony JC, Joseph PN Chapter 23 Waiting lists and medical treatment: Analysis and policies Handbook of Health Economics 2000 North-Holland: Elsevier 1201 1249

[B5] Morris S Elliot B Ma A McConnachie A Rice N Skatun D Sutton M Analysis of consultants’ NHS and private incomes in England in 2003/4 J R Soc Med 2008 14 7 372 380 10.1258/jrsm.2008.080004 18591691 PMC2442143

[B6] Brekke KR Sorgard L Public versus private health care in a national health service Health Econ 2007 14 6 579 601 10.1002/hec.1185 17163459

[B7] Askildsen JEHJ Wages and Work Conditions as Determinants for Physicians’ Work Decisions Working papers no 06/06 in Economics 2006 Norway: University of Bergen

[B8] Van Lerberghe W Conceicao C Van Damme W Ferrinho P When staff is underpaid: dealing with the individual coping strategies of health personnel Bull World Health Organ 2002 14 7 581 584 12163923 PMC2567566

[B9] Bloor K Maynard A Freemantle N Variation in activity rates of consultant surgeons and the influence of reward structures in the English NHS J Health Serv Res Policy 2004 14 2 76 84 10.1258/135581904322987481 15099454

[B10] Johnson N Private Markets in Health and Welfare: An International Perspective 1995 Oxford: Berg Publishers Ltd.

[B11] Ferrinho P Van Lerberghe W da Cruz Gomes A Public and private practice: a balancing act for health staff Bull World Health Organ 1999 14 3 209 10212509 PMC2557616

[B12] Rickman N McGuire A Regulating Providers’ Reimbursement in a Mixed Market for Health Care Scot J Polit Econ 1999 14 1 53 71 10.1111/1467-9485.00120

[B13] Morga A Xavier A Hospital Specialists’ Private Practice and its Impact on the Number of NHS Patients Treated and on the Delay for Elective Surgery Discussion Papers 2001 Heslington, York: Department of Economics, University of York

[B14] Biglaiser G M.C., Moonlighting: public service and private practice RAND Journal of Economics 2007 14 1113 1133 10.1111/j.0741-6261.2007.00128.x

[B15] Ferrinho P Omar MC Fernandes M d J Blaise P Bugalho AM Van Lerberghe W Branding, Substituting, Unnecessary Prescriptions and Pilfering: How Medicines Help Health Personnel to Cope in Cape Verde and Mozambique Unpublished Report 2002 Lisbon: AGO, IMP

[B16] Garcia-Prado A Gonzalez P Whom do physicians work for? An analysis of dual practice in the health sector J Health Polit Policy Law 2011 14 2 265 294 10.1215/03616878-1222721 21543706

[B17] Ferrinho P Ven Lerberghe W Fronteira I Hipolito F Biscaia A Dual practice in the health sector: review of the evidence Hum Resour Health 2004 14 1 14 10.1186/1478-4491-2-14 15509305 PMC529467

[B18] Gruen R Anwar R Begum T Killingsworth JR Normand C Dual job holding practitioners in Bangladesh: an exploration Soc Sci Med 2002 14 2 267 279 10.1016/S0277-9536(01)00026-0 11824931

[B19] Socha KZ Bech M Physician dual practice: a review of literature Health Policy 2011 14 1 1 7 10.1016/j.healthpol.2010.10.017 21094557

[B20] Humphrey C Russell J Motivation and values of hospital consultants in south-east England who work in the national health service and do private practice Soc Sci Med 2004 14 6 1241 1250 10.1016/j.socscimed.2003.12.019 15210095

[B21] OECD Health at a Glance 2011: OECD Indicators 2011 France: OECD Publishing

[B22] Hagen TP Kaarboe OM The Norwegian hospital reform of 2002: central government takes over ownership of public hospitals Health Policy 2006 14 3 320 333 10.1016/j.healthpol.2005.06.014 16099530

[B23] Johannessen KA Hagen TP Variations in labor supply between female and male hospital physicians: results from a modern welfare state Health Policy 2012 14 1 74 82 10.1016/j.healthpol.2012.05.009 22739127

[B24] Sloan FA Physician Supply Behavior in Short Run Ind Labor Relat Rev 1975 14 4 549 569 10.2307/2521651

[B25] Noether M The Growing Supply of Physicians: Has the Market Become More Competitive? J Labor Econ 1986 14 4 503 537 10.1086/298108

[B26] Rizzo JA Blumenthal D Physician labor supply: do income effects matter? J Health Econ 1994 14 4 433 453 10.1016/0167-6296(94)90012-4 10140533

[B27] Baltagi BH Bratberg E Holmas TH A panel data study of physicians’ labor supply: the case of Norway Health Econ 2005 14 10 1035 1045 10.1002/hec.991 15791684

[B28] Akaike H Retrov BN, Csaki F Information theory and an extension of the maximum likelihood principle Second International Symposium on Information Theory 1973 Akademiai Kiado: Budapest 267 281

[B29] Sæther M Physicians’ labour supply: the wage impact on hours and practice combination Labour 2005 14 673 703 10.1111/j.1467-9914.2005.00317.x

[B30] Socha K Bech M The relationship between dual practice and physicians’ work behavior in the public hospitals: Results from the Danish survey, in Institute of Public Health – Health Economics University of Southern Denmark 2011

[B31] Wiley MM The Irish health system: developments in strategy, structure, funding and delivery since 1980 Health Econ 2005 14 Suppl 1 S169 S186 16161189 10.1002/hec.1034

[B32] Jan S Bian T Jumpa M Meng Q Nyazema N Prakongsai P Mills A Dual job holding by public sector health professionals in highly resource-constrained settings: problem or solution? Bull World Health Organ 2005 14 10 771 776 16283054 PMC2626421

[B33] Garcia-Prado A Gonzalez P Policy and regulatory responses to dual practice in the health sector Health Policy 2007 14 2–3 142 152 17449134 10.1016/j.healthpol.2007.03.006

[B34] Mossialos E Allin S Davaki K Analysing the Greek health system: a tale of fragmentation and inertia Health Econ 2005 14 Suppl 1 S151 S168 16161195 10.1002/hec.1033

[B35] Jumpa M Jan S Mills A The role of regulation in influencing income-generating activities among public sector doctors in Peru Hum Resour Health 2007 14 5 10.1186/1478-4491-5-5 17324290 PMC1819388

[B36] Macq J Ferolino P De Brouwere V Van Lerberghe W Managing health services in developing countries: between ethics of the civil servant and the need for moonlighting: managing and moonlighting Human Resources for Health Development Journal 2001 14 17 24

[B37] Gonzalez P Macho-Stadler I A theoretical approach to dual practice regulations in the health sector J Health Econ 2013 14 1 66 87 10.1016/j.jhealeco.2012.08.005 23202256

[B38] EuropeanObservatory., European Observatory on Health Systems Policies Snapshots of health systems: the state of affairs in 16 countries in summer 2004 2004 Copenhagen: WHO Regional Office for Europe on Behalf of the European Observatory on Health Systems and Policies

[B39] Accreditation Council for Graduate Medical Education ACGME Duty Hour Standard 2011 Chicago, IL: ACGME

[B40] Directive E. of the European Parliament and of the Council 2003 Directive 2003/88/EC of the European Parliament and of the Council of 4 November 2003 concerning certain aspects of the organization of working time Official Journal L 299 2003 14 11 9 19

[B41] OECD Health at a Glance 2009 2009 Paris, France: OECD Publisher 2009

